# Poly[μ-aqua-aqua-μ_4_-naphthalene-1,8-dicarboxyl­ato-barium]: a layer structure

**DOI:** 10.1107/S1600536813006259

**Published:** 2013-03-16

**Authors:** Dan Zhao, Fei Fei Li, Peng Liang, Jun-Ran Ren, Shen Qiu

**Affiliations:** a Department of Physics and Chemistry, Henan Polytechnic University, Jiaozuo, Henan 454000, People’s Republic of China

## Abstract

The title compound, [Ba(C_12_H_6_O_4_)(H_2_O)_2_]_*n*_, is represented by a layer-like structure built of BaO_8_ polyhedra. The asymmetric unit contains a Ba^2+^ ion, half a coordinating water mol­ecule and half a μ_4_-bridging naphthalene-1,8-dicarboxyl­ate (1,8-nap) ligand, the whole structure being generated by twofold rotational symmetry. The carboxyl­ate groups of the 1,8-nap ligands act as bridges linking four Ba^2+^ ions, while each Ba^2+^ ion is eight-coordinated by O atoms from four 1,8-nap ligands and two coordinating water mol­ecules. In the crystal, there are O—H⋯O hydrogen bonds involving the water mol­ecules and carboxyl­ate O atoms in the BaO_8_ polyhedra. Each BaO_8_ polyhedron is connected *via* corner-sharing water O atoms or edge-sharing ligand O atoms, forming a sheet parallel to the *bc* plane. These sheets stack along the *a*-axis direction and are connected *via* van der Waals forces only. The naphthalene groups protrude above and below the layers of the BaO_8_ polyhedra and there are voids of *ca* 208 Å^3^ bounded by these groups. No residual electron density was found in this region. The crystal studied was twinned by pseudo-merohedry, with a refined twin component ratio of 0.5261 (1):0.4739 (1).

## Related literature

For other compounds based on 1,8-nap ligands, see: Wen *et al.* (2007 ▶, 2008 ▶); Zhang *et al.* (2008 ▶); Fu *et al.* (2011 ▶).
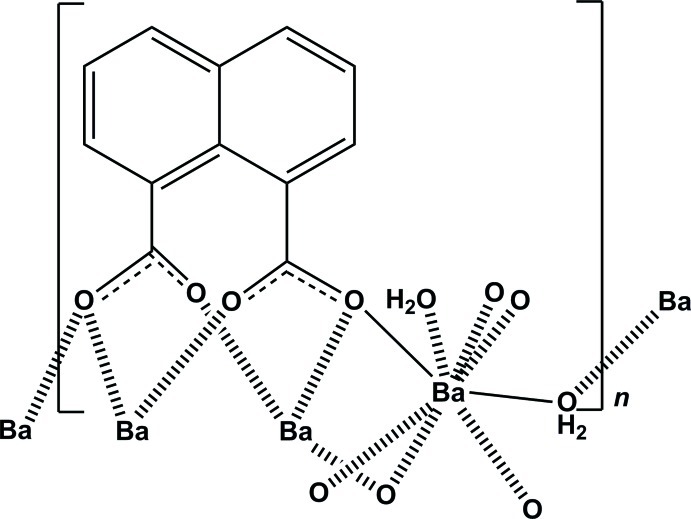


## Experimental

### 

#### Crystal data

[Ba(C_12_H_6_O_4_)(H_2_O)_2_]
*M* *_r_* = 369.52
Orthorhombic, 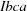

*a* = 8.9643 (11) Å
*b* = 30.539 (6) Å
*c* = 8.9625 (12) Å
*V* = 2453.6 (7) Å^3^
*Z* = 8
Mo *K*α radiation
μ = 3.25 mm^−1^
*T* = 296 K
0.20 × 0.05 × 0.05 mm

#### Data collection

Bruker APEXII CCD area-detector diffractometer
Absorption correction: multi-scan (*SADABS*; Sheldrick, 1996 ▶) *T*_min_ = 0.563, *T*_max_ = 0.855
7968 measured reflections
1511 independent reflections
1344 reflections with *I* > 2σ(*I*)
*R*_int_ = 0.035

#### Refinement

*R*[*F* ^2^ > 2σ(*F* ^2^)] = 0.019
*wR*(*F* ^2^) = 0.043
*S* = 1.05
1511 reflections
85 parameters
H-atom parameters constrained
Δρ_max_ = 0.54 e Å^−3^
Δρ_min_ = −0.57 e Å^−3^

### 

Data collection: *APEX2* (Bruker, 2008 ▶); cell refinement: *SAINT* (Bruker, 2008 ▶); data reduction: *SAINT*; program(s) used to solve structure: *SHELXS97* (Sheldrick, 2008 ▶); program(s) used to refine structure: *SHELXL97* (Sheldrick, 2008 ▶); molecular graphics: *Mercury* (Macrae *et al.*, 2008 ▶); software used to prepare material for publication: *SHELXTL* (Sheldrick, 2008 ▶).

## Supplementary Material

Crystal structure: contains datablock(s) I, global. DOI: 10.1107/S1600536813006259/su2556sup1.cif

Structure factors: contains datablock(s) I. DOI: 10.1107/S1600536813006259/su2556Isup2.hkl

Additional supplementary materials:  crystallographic information; 3D view; checkCIF report

## Figures and Tables

**Table 1:** Hydrogen-bond geometry (Å, °)

*D*—H⋯*A*	*D*—H	H⋯*A*	*D*⋯*A*	*D*—H⋯*A*
O3—H3⋯O2^i^	0.86	2.07	2.777 (2)	140

Symmetry code: (i) 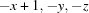.

**Fig. 1.**
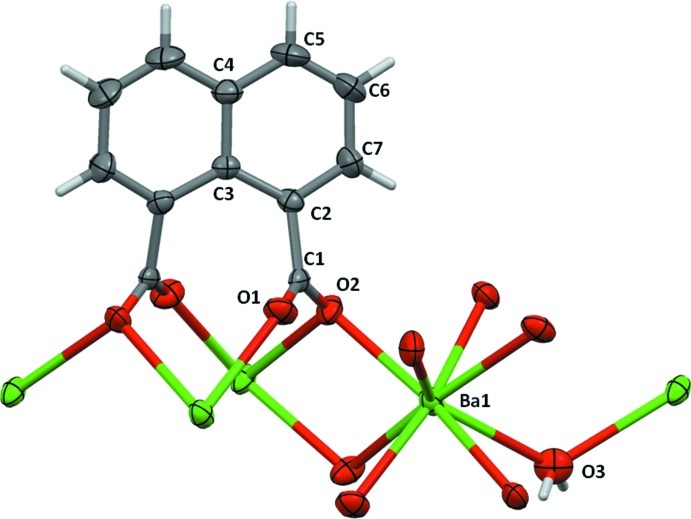
The molecular structure of the title compound showing the coordination environment of the Ba atom. Displacement ellipsoids are drawn at the 50% probability level

**Fig. 2.**
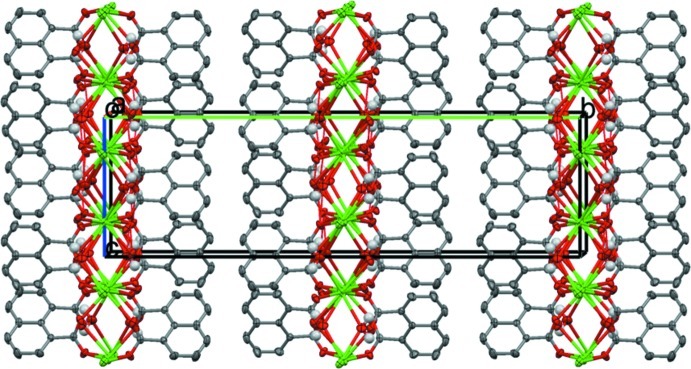
A view along the a axis of the crystal packing of the title compound. The O-H···O hydrogen bonds are shown as red dashed lines (see Table 1 for details).

## supplementary crystallographic information

### Comment

In recent years, supramolecular assembles based on polyoxometalates (POMs) have been intensively investigated in many field such as catalysis, electrical conductivity, and biological chemistry. The ligand naphthalene-1,8-dicarboxylic (1,8-nap) has been used extensively to construct a number of metal organic complexes (Wen *et al., 2007*,2008; Zhang *et al.*, 2008), including the related barium compound Ba(C_12_H_6_O_4_) [Fu *et al.*, 2011]. To prepare a new barium complex incorporating 1,8-nap ligand, we have synthesized the title compound and report herein on its crystal structure.

The title compound is a non-interpenetrating two-dimensional layer-like structure consisting of BaO_8_ clusters, which are similar to the reported compound Ba(C_12_H_6_O_4_) [Fu *et al.*, 2011]. As shown in Fig. 1 the asymmetric unit of the title complex contains one crystallographically independent Ba atom, one coordination water molecule and a half 1,8-nap ligand. Each barium atom is eight-coordinated by O atoms in a square antiprismatic geometry, in which six oxygen atoms come from four 1,8-nap ligands (two of them adopt a chelate connection) and two oxygen atoms come from two coordinated water molecules. The Ba–O bond distances range from 2.723 (2) to 2.8806 (14) Å, in which the Ba1–O3 water bond gives the longest bond distance.

The 1,8-nap ligands are not planar, with the carboxylate groups and the naphthalene ring dihedral angles being 49.0 (3)° and 52.4 (3)°, respectively. The carboxylate groups of the 1,8-nap ligand act as µ_2_-bridges to link four Ba atoms. Furthermore, each BaO_8_ polyhedra is connected *via* corner-sharing H_2_O oxygen atoms or edge-sharing ligand oxygen atoms to form a two-dimensional sheet parallel to the *bc* plane. All Ba atoms in the two-dimensional layer are coplanar, with adjacent Ba···Ba distance of 4.4821 (6), 4.9292 (6) and 5.0972 (6) Å.

By considering the Ba atoms as the nodes, this two-dimensional layered structure can be topologically represented as a 6-connected (3,6) net.

In the crystal, there are O-H···O hydrogen bonds involving the water molecules and the carboxylate O atoms in the BaO_8_ clusters (Fig. 2 and Table 1). There are no π-π stacking interactions, only van der Waals forces are present between the layers that stack along the a direction. The naphthalene groups protrude above and below the layers of the BaO_8_ clusters and there are voids of ca. 208 Å^3^ bounded by these groups. No residual electron density was found in this region.

### Experimental

A mixture of naphthalene-1,8-dicarboxylic (0.2 g), BaCO_3_ (0.05 g) and H_2_O (15 ml) was heated at 443 K for 3 d in a sealed 25 ml Teflon-lined stainless steel vessel under autogenous pressure. After cooling to room temperature at a rate of 20°C h^-1^, colourless prismatic crystals suitable for single-crystal X-ray diffraction analysis were obtained in low yield.

### Refinement

The crystal is a pseudo-merohedral twin, with twin law (00-1, 0-10, -100) giving an ca. 1:1 ratio of twin moieties [refined BASF value = 0.5261 (1)]. The C-bound H atoms were positioned geometrically and refined with a riding model: C—H = 0.93 Å with *U*_iso_(H) = 1.2U_eq_(C). The water H atoms were located in difference Fourier maps and refined initially with distance restraints: O–H = 0.86 Å, then as riding atoms with *U*_iso_(H) = 1.2U_eq_(O).

### Crystal data

**Table:**

[Ba(C_12_H_6_O_4_)(H_2_O)_2_]	*F*(000) = 1408
*M**_r_* = 369.52	*D*_x_ = 2.001 Mg m^−^^3^
Orthorhombic, *I**b**c**a*	Mo *K*α radiation, λ = 0.71073 Å
Hall symbol: -I 2b 2c	Cell parameters from 2836 reflections
*a* = 8.9643 (11) Å	θ = 2.7–27.1°
*b* = 30.539 (6) Å	µ = 3.25 mm^−^^1^
*c* = 8.9625 (12) Å	*T* = 296 K
*V* = 2453.6 (7) Å^3^	Prism, colourless
*Z* = 8	0.20 × 0.05 × 0.05 mm

### Data collection

**Table:**

Bruker APEXII CCD area-detector diffractometer	1511 independent reflections
Radiation source: fine-focus sealed tube	1344 reflections with *I* > 2σ(*I*)
Graphite monochromator	*R*_int_ = 0.035
Detector resolution: 83.33 pixels mm^-1^	θ_max_ = 28.4°, θ_min_ = 1.3°
ω scans	*h* = −11→5
Absorption correction: multi-scan (*SADABS*; Sheldrick, 1996)	*k* = −40→38
*T*_min_ = 0.563, *T*_max_ = 0.855	*l* = −11→10
7968 measured reflections	

### Refinement

**Table:**

Refinement on *F*^2^	Primary atom site location: structure-invariant direct methods
Least-squares matrix: full	Secondary atom site location: difference Fourier map
*R*[*F*^2^ > 2σ(*F*^2^)] = 0.019	Hydrogen site location: inferred from neighbouring sites
*wR*(*F*^2^) = 0.043	H-atom parameters constrained
*S* = 1.05	*w* = 1/[σ^2^(*F*_o_^2^) + (0.0197*P*)^2^ + 0.1976*P*] where *P* = (*F*_o_^2^ + 2*F*_c_^2^)/3
1511 reflections	(Δ/σ)_max_ = 0.001
85 parameters	Δρ_max_ = 0.54 e Å^−^^3^
0 restraints	Δρ_min_ = −0.57 e Å^−^^3^

### Special details

**Table:**

Geometry. All e.s.d.'s (except the e.s.d. in the dihedral angle between two l.s. planes) are estimated using the full covariance matrix. The cell e.s.d.'s are taken into account individually in the estimation of e.s.d.'s in distances, angles and torsion angles; correlations between e.s.d.'s in cell parameters are only used when they are defined by crystal symmetry. An approximate (isotropic) treatment of cell e.s.d.'s is used for estimating e.s.d.'s involving l.s. planes.
Refinement. Refinement of *F*^2^ against ALL reflections. The weighted *R*-factor *wR* and goodness of fit *S* are based on *F*^2^, conventional *R*-factors *R* are based on *F*, with *F* set to zero for negative *F*^2^. The threshold expression of *F*^2^ > σ(*F*^2^) is used only for calculating *R*-factors(gt) *etc*. and is not relevant to the choice of reflections for refinement. *R*-factors based on *F*^2^ are statistically about twice as large as those based on *F*, and *R*- factors based on ALL data will be even larger.

### Fractional atomic coordinates and isotropic or equivalent isotropic displacement parameters (Å^2^)

**Table:**

	*x*	*y*	*z*	*U*_iso_*/*U*_eq_	
Ba1	0.61452 (2)	0.0000	0.2500	0.02892 (7)	
O1	0.4190 (2)	0.05878 (6)	0.5240 (3)	0.0360 (5)	
C1	0.3754 (3)	0.07360 (9)	0.4035 (4)	0.0236 (6)	
O2	0.3671 (2)	0.05119 (7)	0.2858 (2)	0.0341 (5)	
C2	0.3410 (3)	0.12190 (9)	0.3935 (3)	0.0253 (6)	
O3	0.7500	−0.04396 (9)	0.0000	0.0418 (8)	
H3	0.7197	−0.0594	−0.0742	0.050*	
C3	0.2500	0.14342 (12)	0.5000	0.0239 (8)	
C4	0.2500	0.19041 (12)	0.5000	0.0324 (10)	
C5	0.3323 (4)	0.21277 (11)	0.3902 (5)	0.0486 (10)	
H5	0.3325	0.2432	0.3899	0.058*	
C6	0.4105 (4)	0.19121 (11)	0.2860 (4)	0.0527 (10)	
H6	0.4610	0.2067	0.2123	0.063*	
C7	0.4163 (4)	0.14507 (10)	0.2877 (3)	0.0386 (8)	
H7	0.4720	0.1303	0.2160	0.046*	

### Atomic displacement parameters (Å^2^)

**Table:**

	*U*^11^	*U*^22^	*U*^33^	*U*^12^	*U*^13^	*U*^23^
Ba1	0.01894 (11)	0.03182 (12)	0.03599 (14)	0.000	0.000	0.00999 (13)
O1	0.0321 (12)	0.0315 (11)	0.0445 (13)	0.0027 (9)	−0.0065 (10)	0.0124 (10)
C1	0.0140 (12)	0.0213 (14)	0.0356 (16)	−0.0012 (10)	0.0042 (11)	−0.0009 (12)
O2	0.0330 (11)	0.0314 (11)	0.0380 (14)	−0.0029 (8)	0.0096 (8)	−0.0132 (9)
C2	0.0279 (15)	0.0225 (15)	0.0255 (15)	−0.0033 (12)	−0.0016 (11)	−0.0004 (12)
O3	0.0412 (19)	0.0472 (18)	0.037 (2)	0.000	−0.0162 (15)	0.000
C3	0.025 (2)	0.0220 (19)	0.025 (2)	0.000	−0.0046 (16)	0.000
C4	0.034 (2)	0.022 (2)	0.041 (3)	0.000	−0.0010 (19)	0.000
C5	0.061 (2)	0.0203 (17)	0.065 (3)	−0.0016 (16)	0.0042 (19)	0.0087 (16)
C6	0.067 (2)	0.0326 (18)	0.058 (3)	−0.0034 (16)	0.0213 (19)	0.0164 (15)
C7	0.0463 (18)	0.0366 (17)	0.033 (2)	−0.0007 (14)	0.0128 (13)	0.0060 (13)

### Geometric parameters (Å, º)

**Table:**

Ba1—O1^i^	2.723 (2)	C2—C7	1.362 (4)
Ba1—O1^ii^	2.723 (2)	C2—C3	1.417 (3)
Ba1—O2^iii^	2.7324 (19)	O3—Ba1^viii^	2.8806 (14)
Ba1—O2	2.7324 (19)	O3—H3	0.8587
Ba1—O2^iv^	2.7703 (19)	C3—C2^ix^	1.417 (3)
Ba1—O2^v^	2.7703 (19)	C3—C4	1.435 (5)
Ba1—O3^vi^	2.8806 (14)	C4—C5^ix^	1.407 (4)
Ba1—O3	2.8806 (14)	C4—C5	1.407 (4)
O1—C1	1.234 (4)	C5—C6	1.341 (5)
O1—Ba1^ii^	2.723 (2)	C5—H5	0.9300
C1—O2	1.259 (4)	C6—C7	1.410 (4)
C1—C2	1.509 (4)	C6—H6	0.9300
O2—Ba1^vii^	2.7703 (19)	C7—H7	0.9300
			
O1^i^—Ba1—O1^ii^	167.34 (9)	O2^iv^—Ba1—Ba1^vii^	144.82 (4)
O1^i^—Ba1—O2^iii^	101.55 (6)	O2^v^—Ba1—Ba1^vii^	144.82 (4)
O1^ii^—Ba1—O2^iii^	67.71 (6)	O3^vi^—Ba1—Ba1^vii^	114.937 (13)
O1^i^—Ba1—O2	67.71 (6)	O3—Ba1—Ba1^vii^	114.937 (13)
O1^ii^—Ba1—O2	101.55 (6)	C1^iii^—Ba1—Ba1^vii^	50.87 (4)
O2^iii^—Ba1—O2	71.48 (8)	C1—Ba1—Ba1^vii^	50.87 (4)
O1^i^—Ba1—O2^iv^	68.40 (6)	O1^i^—Ba1—Ba1^iv^	96.33 (4)
O1^ii^—Ba1—O2^iv^	123.25 (6)	O1^ii^—Ba1—Ba1^iv^	96.33 (4)
O2^iii^—Ba1—O2^iv^	166.59 (9)	O2^iii^—Ba1—Ba1^iv^	144.26 (4)
O2—Ba1—O2^iv^	110.74 (7)	O2—Ba1—Ba1^iv^	144.26 (4)
O1^i^—Ba1—O2^v^	123.25 (6)	O2^iv^—Ba1—Ba1^iv^	35.18 (4)
O1^ii^—Ba1—O2^v^	68.40 (6)	O2^v^—Ba1—Ba1^iv^	35.18 (4)
O2^iii^—Ba1—O2^v^	110.74 (7)	O3^vi^—Ba1—Ba1^iv^	65.063 (13)
O2—Ba1—O2^v^	166.59 (9)	O3—Ba1—Ba1^iv^	65.063 (13)
O2^iv^—Ba1—O2^v^	70.35 (8)	C1^iii^—Ba1—Ba1^iv^	129.13 (4)
O1^i^—Ba1—O3^vi^	108.54 (7)	C1—Ba1—Ba1^iv^	129.13 (4)
O1^ii^—Ba1—O3^vi^	77.00 (7)	Ba1^vii^—Ba1—Ba1^iv^	180.0
O2^iii^—Ba1—O3^vi^	134.45 (5)	C1—O1—Ba1^ii^	149.05 (19)
O2—Ba1—O3^vi^	89.09 (5)	O1—C1—O2	123.5 (3)
O2^iv^—Ba1—O3^vi^	58.83 (5)	O1—C1—C2	118.3 (3)
O2^v^—Ba1—O3^vi^	80.11 (6)	O2—C1—C2	118.0 (3)
O1^i^—Ba1—O3	77.00 (7)	O1—C1—Ba1	84.93 (16)
O1^ii^—Ba1—O3	108.54 (7)	O2—C1—Ba1	48.58 (13)
O2^iii^—Ba1—O3	89.09 (5)	C2—C1—Ba1	138.71 (18)
O2—Ba1—O3	134.45 (5)	C1—O2—Ba1	111.20 (16)
O2^iv^—Ba1—O3	80.11 (6)	C1—O2—Ba1^vii^	116.85 (16)
O2^v^—Ba1—O3	58.83 (5)	Ba1—O2—Ba1^vii^	109.08 (7)
O3^vi^—Ba1—O3	130.13 (3)	C7—C2—C3	120.9 (3)
O1^i^—Ba1—C1^iii^	93.75 (7)	C7—C2—C1	116.6 (3)
O1^ii^—Ba1—C1^iii^	78.20 (7)	C3—C2—C1	122.1 (3)
O2^iii^—Ba1—C1^iii^	20.22 (6)	Ba1^viii^—O3—Ba1	124.44 (10)
O2—Ba1—C1^iii^	85.06 (6)	Ba1^viii^—O3—H3	77.7
O2^iv^—Ba1—C1^iii^	147.38 (7)	Ba1—O3—H3	136.4
O2^v^—Ba1—C1^iii^	100.91 (6)	C2—C3—C2^ix^	124.8 (3)
O3^vi^—Ba1—C1^iii^	152.80 (6)	C2—C3—C4	117.62 (17)
O3—Ba1—C1^iii^	69.07 (6)	C2^ix^—C3—C4	117.62 (17)
O1^i^—Ba1—C1	78.20 (7)	C5^ix^—C4—C5	121.9 (4)
O1^ii^—Ba1—C1	93.75 (7)	C5^ix^—C4—C3	119.0 (2)
O2^iii^—Ba1—C1	85.06 (6)	C5—C4—C3	119.0 (2)
O2—Ba1—C1	20.22 (6)	C6—C5—C4	121.6 (3)
O2^iv^—Ba1—C1	100.91 (6)	C6—C5—H5	119.2
O2^v^—Ba1—C1	147.38 (7)	C4—C5—H5	119.2
O3^vi^—Ba1—C1	69.07 (6)	C5—C6—C7	120.2 (3)
O3—Ba1—C1	152.80 (6)	C5—C6—H6	119.9
C1^iii^—Ba1—C1	101.74 (9)	C7—C6—H6	119.9
O1^i^—Ba1—Ba1^vii^	83.67 (4)	C2—C7—C6	120.5 (3)
O1^ii^—Ba1—Ba1^vii^	83.67 (4)	C2—C7—H7	119.7
O2^iii^—Ba1—Ba1^vii^	35.74 (4)	C6—C7—H7	119.7
O2—Ba1—Ba1^vii^	35.74 (4)		

Symmetry codes: (i) −*x*+1, *y*, *z*−1/2; (ii) −*x*+1, −*y*, −*z*+1; (iii) *x*, −*y*, −*z*+1/2; (iv) *x*+1/2, *y*, −*z*+1/2; (v) *x*+1/2, −*y*, *z*; (vi) −*x*+3/2, −*y*, *z*+1/2; (vii) *x*−1/2, *y*, −*z*+1/2; (viii) −*x*+3/2, −*y*, *z*−1/2; (ix) −*x*+1/2, *y*, −*z*+1.

### Hydrogen-bond geometry (Å, º)

**Table:**

*D*—H···*A*	*D*—H	H···*A*	*D*···*A*	*D*—H···*A*
O3—H3···O2^x^	0.86	2.07	2.777 (2)	140

Symmetry code: (x) −*x*+1, −*y*, −*z*.
